# An integrative analysis of the lncRNA-miRNA-mRNA competitive endogenous RNA network reveals potential mechanisms in the murine hair follicle cycle

**DOI:** 10.3389/fgene.2022.931797

**Published:** 2022-10-25

**Authors:** Yuxin Ding, Yuhong Chen, Xiaoshuang Yang, Piaopiao Xu, Jing Jing, Yujie Miao, Meiqi Mao, Jiali Xu, Xianjie Wu, Zhongfa Lu

**Affiliations:** Department of Dermatology, The Second Affiliated Hospital, School of Medicine, Zhejiang University, Hangzhou, China

**Keywords:** hair follicle cycle, murine hair follicle, androgenetic alopecia, noncoding RNA, ceRNA

## Abstract

Alopecia is a common progressive disorder associated with abnormalities of the hair follicle cycle. Hair follicles undergo cyclic phases of hair growth (anagen), regression (catagen), and rest (telogen), which are precisely regulated by various mechanisms. However, the specific mechanism associated with hair follicle cycling, which includes noncoding RNAs and regulation of competitive endogenous RNA (ceRNA) network, is still unclear. We obtained data from publicly available databases and performed real-time quantitative polymerase chain reaction validations. These analyses revealed an increase in the expression of miRNAs and a decrease in the expression of target mRNAs and lncRNAs from the anagen to telogen phase of the murine hair follicle cycle. Subsequently, we constructed the ceRNA networks and investigated their functions using enrichment analysis. Furthermore, the androgenetic alopecia (AGA) microarray data analysis revealed that several novel alopecia-related genes were identified in the ceRNA networks. Lastly, GSPT1 expression was detected using immunohistochemistry. Our analysis revealed 11 miRNAs (miR-148a-3p, miR-146a-5p, miR-200a-3p, miR-30e-5p, miR-30a-5p, miR-27a-3p, miR-143-3p, miR-27b-3p, miR-126a-3p, miR-378a-3p, and miR-22-3p), 9 target mRNAs (*Atp6v1a*, *Cdkn1a*, *Gadd45a*, *Gspt1*, *Mafb*, *Mitf*, *Notch1*, *Plk2*, and *Slc7a5*), and 2 target lncRNAs (Neat1 and Tug1) were differentially expressed in hair follicle cycling. The ceRNA networks were made of 12 interactive miRNA-mRNA pairs and 13 miRNA-lncRNA pairs. The functional enrichment analysis revealed the enrichment of hair growth–related signaling pathways. Additionally, GSPT1 was downregulated in androgenetic alopecia patients, possibly associated with alopecia progression. The ceRNA network identified by our analysis could be involved in regulating the hair follicle cycle.

## Introduction

Hair loss, also known as alopecia, is a commonly diagnosed disorder which severely affects the mental health of patients (Hunt and McHale, 2005). The primary causes of hair follicle cycle abnormalities that lead to alopecia include genetics, immune dysregulation, endocrine disturbance, and aging (Ebling, 1976; Salim and Kamalasanan, 2020). Hair follicles are regenerative organs that undergo a cyclic phase of hair growth (anagen), regression with programmed cell death (catagen), and relative rest phase (telogen) (Paus and Cotsarelis, 1999). The key to treatment for alopecia is driving regenerative hair cycles and reestablishing normal hair cycles (Price, 1999; Salim and Kamalasanan, 2020). Human hair follicles have unsynchronized cycles, hence, it is challenging to study the molecular mechanisms of the hair follicle cycle (Stenn and Paus, 2001). On the contrary, the murine hair follicle cycle is synchronized through the first two postnatal hair follicle cycles and could serve as a good model to study hair follicle cycling (Muller-Rover et al., 2001). The hair follicle cycles are precisely regulated by various signaling pathways, such as Wnt/β-Catenin, bone morphogenetic protein, fibroblast growth factor, and sonic hedgehog signaling (Huelsken et al., 2001; Plikus et al., 2008; Hsu et al., 2014; Harshuk-Shabso et al., 2020). However, the precise mechanism and signaling networks associated with the hair follicle cycle are still unclear.

MicroRNAs (miRNAs) are evolutionarily conserved noncoding ∼23-nucleotides-long single-stranded RNA. They play a role in the posttranscriptional regulation of protein-coding genes (Li and Rana, 2014). Mounting evidence suggests that miRNAs regulate the hair follicle cycle (Yi et al., 2006; Botchkareva, 2012; Andl and Botchkareva, 2015). Several specific miRNAs, which include miR-22, miR-29a, miR-31, and miR-214, are dynamically expressed at different stages of the murine hair follicle cycle and regulate hair growth (Mardaryev et al., 2010; Ahmed et al., 2014; Yuan et al., 2015; Ge et al., 2019). Long noncoding RNAs (lncRNAs) are 200-nucleotides-long nonprotein-coding RNA transcripts (Matsui and Corey, 2017). Functional studies have indicated that lncRNAs are involved in diverse biological processes (BP) using multiple mechanisms (Statello et al., 2021). However, the role of lncRNAs in the hair follicle cycle is still unclear. Recently studies have shown that some lncRNAs, such as lncRNA5322, LNC_002919, and H19, may be associated with hair growth and hair cycle (Zhu et al., 2018; Cai et al., 2019; Zhao et al., 2019). As per the competing endogenous RNA (ceRNA) hypothesis, lncRNA acts as a sponge for miRNAs, indirectly preventing the degradation of the target mRNAs by the shared miRNAs (Salmena et al., 2011). Wang et al. (2017) analyzed and constructed ceRNA regulatory networks in the cashmere goat hair follicle cycle. Zhao et al. (2019) analyzed ceRNA in the Angora rabbit hair cycle. These studies are closely associated with animal husbandry and have economic value. However, the role of ceRNA networks in regulating hair cycle and alopecia is yet to be explored.

High-throughput techniques such as microarray and RNA sequencing have made transcriptomic analysis relatively easy and have increased the accuracy of the analysis. Various studies have used high-throughput screening techniques and generated a large volume of highly sensitive data, enhancing our understanding of hair cycle regulation (Bao et al., 2017; Sakamoto et al., 2021). However, the major limitation of these high-throughput screening techniques is the high rate of false-positive results. Thus, experimental validation using quantitative real-time polymerase chain reaction (RT-qPCR) should be carried out to validate high-throughput screening results.

**FIGURE 1:**
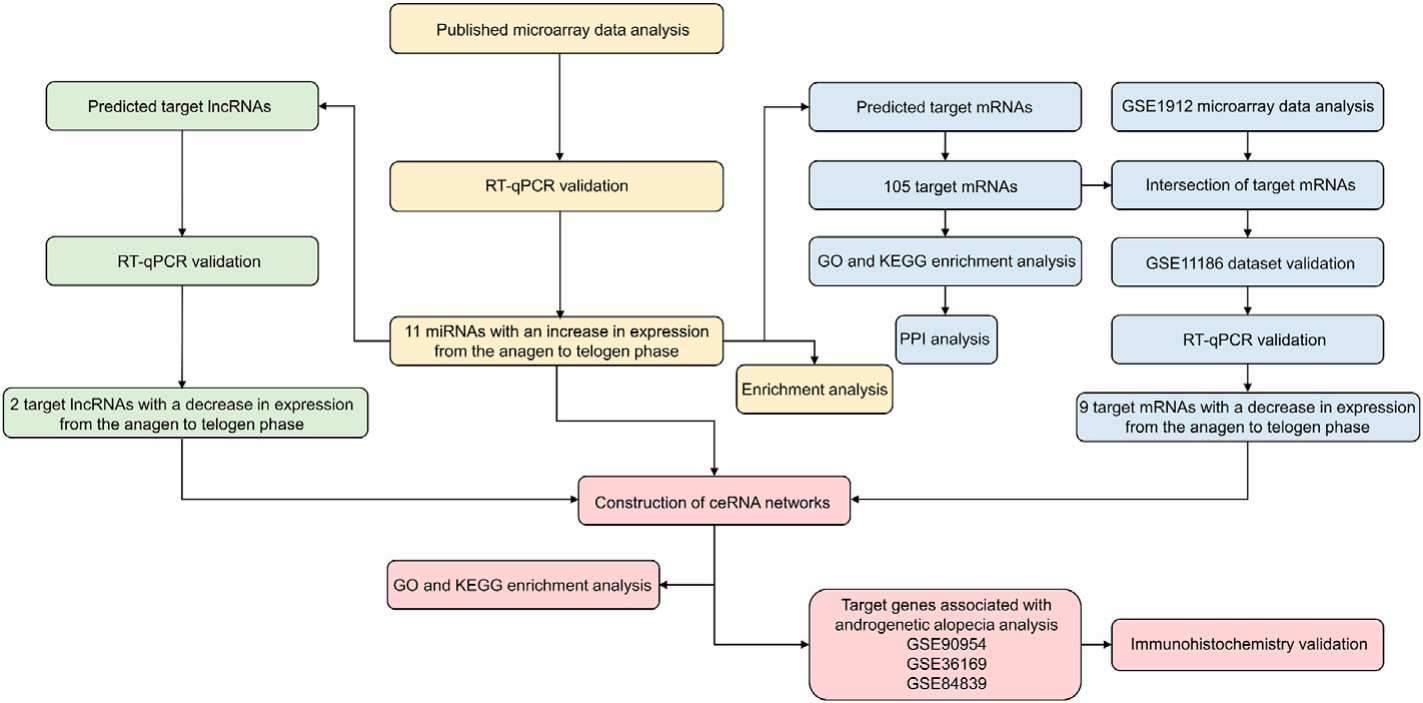
Research flowchart.

In this study, we used multiple publicly available high-quality microarray data sets to analyze the murine hair follicle cycle. We first identified and experimentally validated 11 miRNAs with an increase in expression during the transition from the anagen to telogen phase in the murine hair follicle cycle. Subsequently, the analysis and validation of the target genes, which included their mRNAs and lncRNAs, which may be negatively regulated by the screened miRNAs, were carried out. Finally, we established the ceRNA networks of the murine hair follicle cycle (Figure 1). Our study has demonstrated the first ceRNA networks in the mouse hair follicle cycle, thereby strengthening our understanding of hair follicle cycle regulation. We have also identified genes that could be involved in alopecia.

## Materials and methods

### Mice

All animal studies were approved by the Animal Ethics Committee of the Second Affiliated Hospital, School of Medicine, Zhejiang University (approval number #2019-040). Dorsal skin tissues were collected from neonatal C57BL/6 mice at postnatal day 14 (P14, anagen), P17 (catagen), and P23 (telogen). Three mice were used per time point. Skin tissues were immediately frozen in liquid nitrogen or fixed with 4% paraformaldehyde for hematoxylin and eosin (HE) staining.

### Human scalp samples

Human scalp samples were obtained from three androgenetic alopecia (AGA) patients and five healthy donors (male, aged 25–30 years, no finasteride or minoxidil taking) undergoing cosmetic surgery. The study was approved by the local ethics committee, and informed consent was obtained from the participants (approval number #2021-0173).

### Data set selection

Published microarray data were used to obtain the expression values for differentially expressed miRNAs at different stages of the hair follicle cycle (Mardaryev et al., 2010). Gene expression data of mouse hair follicle cycle were retrieved from databases such as Gene Expression Omnibus (GEO). Data sets from GSE1912 (which included three anagen and three telogen samples) and GSE11186 (which included 12 anagen, 3 catagen, and 7 telogen samples) were selected for subsequent analysis (Lin et al., 2004; Lin et al., 2009). Human AGA mRNA and lncRNA microarray data were downloaded from GEO with the accession number GSE90594 (which included 14 AGA and 14 healthy samples), GSE36169 (which included five bald samples and five paired haired samples), and GSE84839 (which included three bald samples and paired three haired samples) (Garza et al., 2012; Bao et al., 2017; Michel et al., 2017). All samples used in the study are listed in Supplementary Table S1.

### Differential expression analysis

Differentially expressed miRNAs with *p* < 0.001 in the published microarray data were selected for further analysis. The fold change (FC) of miRNA was calculated by comparing the expression values of miRNA in the telogen or catagen group to the expression values of miRNA in the anagen control group. An increase in the expression of miRNAs was considered significant from the anagen to telogen phase if both log_2_ FC > 1. The expression data from the data set GSE1912 were used to identify differentially expressed mRNAs between the telogen and anagen stages. The “affy” and “limma” packages were used to normalize the expression data and perform differential expression analysis. *p* < 0.05 for *t*-test and FC > 2 or FC < 1/2 were chosen as the thresholds for differential expression. The expression data from data set GSE11186 were used as a validation data set. The expression data were extracted from the Series Matrix file using the log_2_ standardized method. The Graphpad software was used for statistical analysis and to create line charts. The “ggplot2” package was used to create volcano plots and Venn diagrams. The expression heatmap was constructed using the R package ComplexHeatmap. Human AGA microarray mRNA and lncRNA expression data were analyzed using the *t*-test or paired *t*-test. *p* < 0.05 was considered statistically significant. The graphics were created using ggplot.

### Functional enrichment analysis and construction of protein–protein interaction network

GO enrichment analysis was performed using miRPathDB v2.0, a bioinformatics tool that analyzes pathways enriched by various levels of miRNA interactions (Kehl et al., 2020). To ensure that the analysis was biologically relevant, the heatmap generated was based on pathways enriched by all differentially expressed miRNAs. The Kyoto Encyclopedia of Genes and Genomes (KEGG) pathway enrichment analysis of miRNAs was performed using FunRich (v3.1.3), a stand-alone functional enrichment analysis tool (Fonseka et al., 2021). *p*<0.05 was considered statistically significant.

Functional enrichment analysis of mRNAs was performed using Database for Annotation, Visualization, and Integration Discovery (DAVID) version 2021. The GO terms and KEGG pathway enrichment analyses were considered significant if *p* < 0.05 and FDR *q* < 0.05. The enrichment analysis results were plotted using http://www.bioinformatics.com.cn, a free web-based platform for data analysis and visualization. PPI networks were constructed and modularized using the Metascape tool (Zhou et al., 2019).

### Prediction of target mRNAs and lncRNAs

Databases such as the TargetScan v8.0, miRDB, and PITA were used to predict miRNAs that were complementary to 3′-untranslated regions in mRNAs (Kertesz et al., 2007; McGeary et al., 2019; Chen and Wang, 2020). Previously published studies were reviewed to identify experimental evidence on the target mRNA interactions predicted by these databases. To reduce false positives, miRNA-mRNA interactions predicted using computational methods were excluded from the analysis. The Encyclopedia of RNA Interactomes (ENCORI) database was used to identify the target lncRNAs (Li et al., 2014). The subcellular localization of lncRNAs was determined using databases such as RNALocate v2.0 and lncATLAS (Mas-Ponte et al., 2017; Cui et al., 2022). lncRNAs localized in the nucleus were excluded because they did not align with the ceRNA hypothesis (Salmena et al., 2011). In addition, the miRNA-lncRNA interactions obtained from previously published studies and validated experimentally were considered. The Cytoscape software v3.9.1 was used to create interaction networks.

### Quantitative real-time polymerase chain reaction

Total RNA was isolated from skin samples using TRIzol reagent (Thermo Fisher Scientific, Braunschweig, Germany) according to the manufacturer’s protocol. 1 μg RNA was used to synthesize complementary DNA (cDNA) using the PrimeScript RT Reagent Kit (RR037A, Takara, Shiga, Japan) or Mir-X miRNA First-Strand Synthesis Kit (638315, Takara). The primer sequences are shown in Supplementary Table S2. RT-qPCR was performed using TB Green^®^ Premix Ex Taq™ II (RR820A, Takara) according to the manufacturer’s instructions. Briefly, RT-qPCR was performed with 10 μl TB Green Premix Ex Taq II, gene-specific primers (400 nM final concentration of forward and reverse primers), 2 μl cDNA, and 6.4 μl ddH_2_O. All RT-qPCR reactions were performed in triplicate. The cycling conditions for RT-qPCR are as follows: step 1: denaturation at 95°C for 30 s and step 2: 40 cycles at 95°C for 5 s and 60°C for 34 s. *GAPDH/U6* was used as the endogenous control. The relative expression of the genes was calculated using the 2^−ΔΔCt^ method.

### Immunohistochemistry

5 μm thick serial sections of paraffin-embedded human scalp tissues were made, and immunohistochemistry was performed using anti-GSPT1 (1:200 dilution, 10763-1-AP, Proteintech, IL, United States). The negative controls were sectioned without the primary antibody. The slides were visualized under a light microscope (Leica, Wetzlar, Germany), and the images were captured. The clinical information was blinded, and two independent pathologists evaluated the slides.

### Statistical analysis

Statistical analyses were completed using R (v3.6.3) software or bioinformatics tools mentioned previously. The data were compared using paired Student’s *t*-tests, unpaired two-tailed *t*-tests, and one-way analysis of variance. The results are represented as mean ± standard deviation.

## Results

### Screening for miRNAs with increase in expression from anagen to telogen phases

Previously published microarray data were used to obtain a list of differentially expressed miRNAs in the mouse hair follicle cycle. The miRNAs with *p* values < 0.001 were selected for further analysis. To identify the highly expressed miRNAs during the telogen phase, we calculated the ratio of expression value of all the selected miRNA between the telogen and anagen phases. The ratio of the miRNA expression value at the catagen phase to miRNA expression value at the anagen phase was also calculated. The miRNA expression was considered to be enhanced from the anagen phase to the telogen phase in the hair follicle cycle, when both ratio log_2_ FC > 1. Eleven miRNAs were identified based on these criteria: miR-148a-3p, miR-146a-5p, miR-200a-3p, miR-30e-5p, miR-30a-5p, miR-27a-3p, miR-143-3p, miR-27b-3p, miR-126a-3p, miR-378a-3p, and miR-22-3p (Table 1).

**TABLE 1:** A set of miRNAs with increasing expression from the anagen to telogen phase in murine hair follicle cycle.

miRNA	Anagen	Catagen	Telogen	*p* value	log2 FC (T/A)	log2 FC (C/A)
miR-148a-3p	250	854	2796	2.48E-12	3.48	1.77
miR-146a-5p	78	169	678	4.44E-16	3.12	1.12
miR-200a-3p	144	842	809	4.34E-13	2.49	2.55
miR-30e-5p	95	244	514	2.11E-13	2.44	1.36
miR-30a-5p	445	1226	2074	6.16E-13	2.22	1.46
miR-27a-3p	1744	5366	7887	1.13E-11	2.18	1.62
miR-143-3p	941	2705	3587	4.02E-13	1.93	1.52
miR-27b-3p	2699	8388	9501	1.86E-12	1.82	1.64
miR-126a-3p	2239	5403	7564	8.03E-13	1.76	1.27
miR-378a-3p	215	462	511	1.21E-14	1.25	1.10
miR-22-3p	231	484	535	4.38E-05	1.21	1.07

A, anagen; C, catagen; T, telogen; FC, fold change.

**FIGURE 2:**
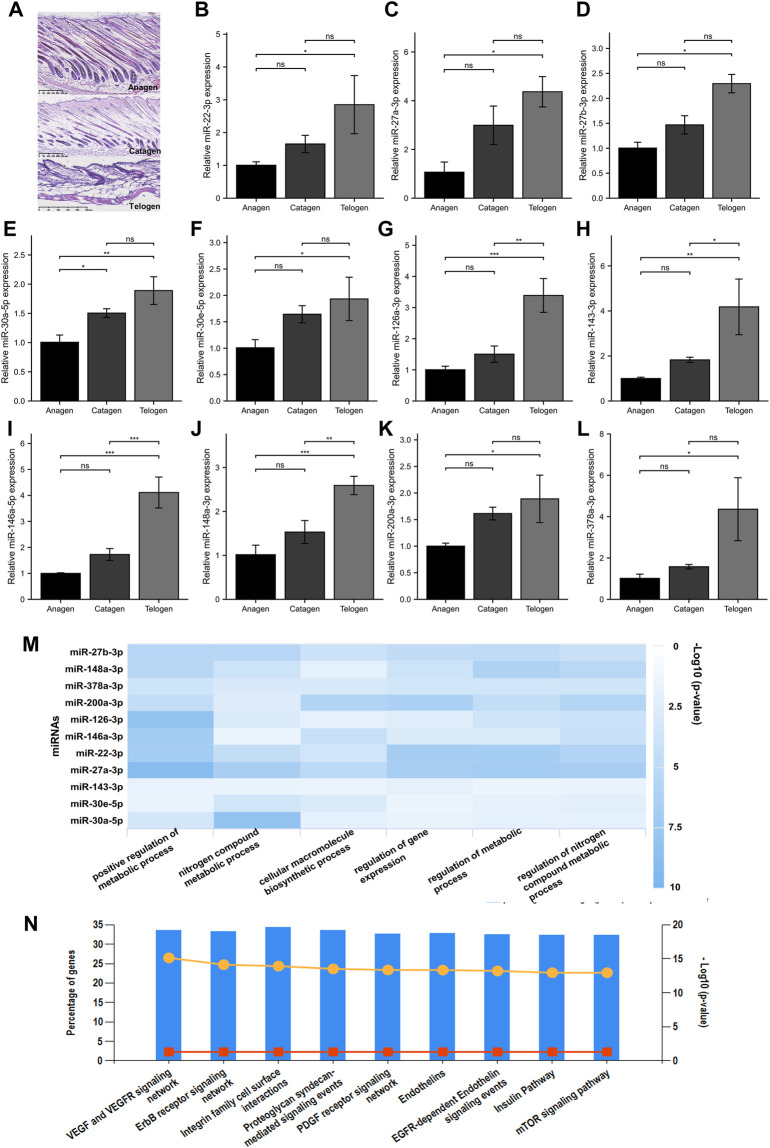
Screening for miRNA with increased expression from the anagen to telogen phase in murine hair follicle cycle. **(A)** HE staining of murine hair follicle morphology shows anagen (P14), catagen (P17), and telogen (P23). **(B–L)** Validation of screened miRNAs using RT-qPCR. The expressions of miR-22-3p, miR-27a-3p, miR-27b-3p, miR-30a-5p, miR-30e-5p, miR-126a-3p, miR-143-3p, miR-146a-5p, miR-148a-3p, miR-200a-3p, and miR-378a-3p at different stages of the hair follicle cycle. **(M)** The GO biological processes enrichment analysis of 11 screened miRNAs. **(N)** The KEGG pathway enrichment analysis of 11 screened miRNAs. ns, *p* ≥ 0.05; *, *p* < 0.05; **, *p* < 0.01; ***, *p* < 0.001. HE, hematoxylin and eosin; *p*, postnatal; GO, Gene Ontology; KEGG, Kyoto Encyclopedia of Genes and Genomes.

Based on the comprehensive guide for murine hair follicles at different stages of the cycle, the dorsal skin samples at the anagen (P14), catagen (P17), and telogen (P23) phases were collected from C57BL/6 mice with a highly synchronized hair cycle. The morphology of the hair follicles was evaluated using HE staining. Figure 2A shows the different stages of the hair follicle cycle. The screened miRNAs were validated using RT-qPCR. Eleven miRNAs were significantly differentially expressed between the telogen and anagen phases. Furthermore, an increasing trend in miRNA expression was observed from the anagen to telogen phases (Figure 2B–L).

To explore the biological functions of the identified miRNAs, the bioinformatic tools miRPathDB (v2.0) and FunRich (v3.1.3) were used to perform GO and KEGG pathway enrichment analyses, respectively. The GO enrichment analysis revealed that the BP were significantly enriched when the 11 miRNAs were regulated by the metabolic process, cellular macromolecule biosynthetic process, and regulation of gene expression (Figure 2M). The KEGG pathway enrichment analysis revealed that various hair growth and cycling–related pathways were enriched, such as the VEGF and VEGFR signaling pathways, ErbB receptor signaling pathway, integrin family cell surface interactions, and mTOR signaling pathway (Figure 2N).

### Screening for target mRNA with decrease in expression from anagen to telogen phases

**FIGURE 3:**
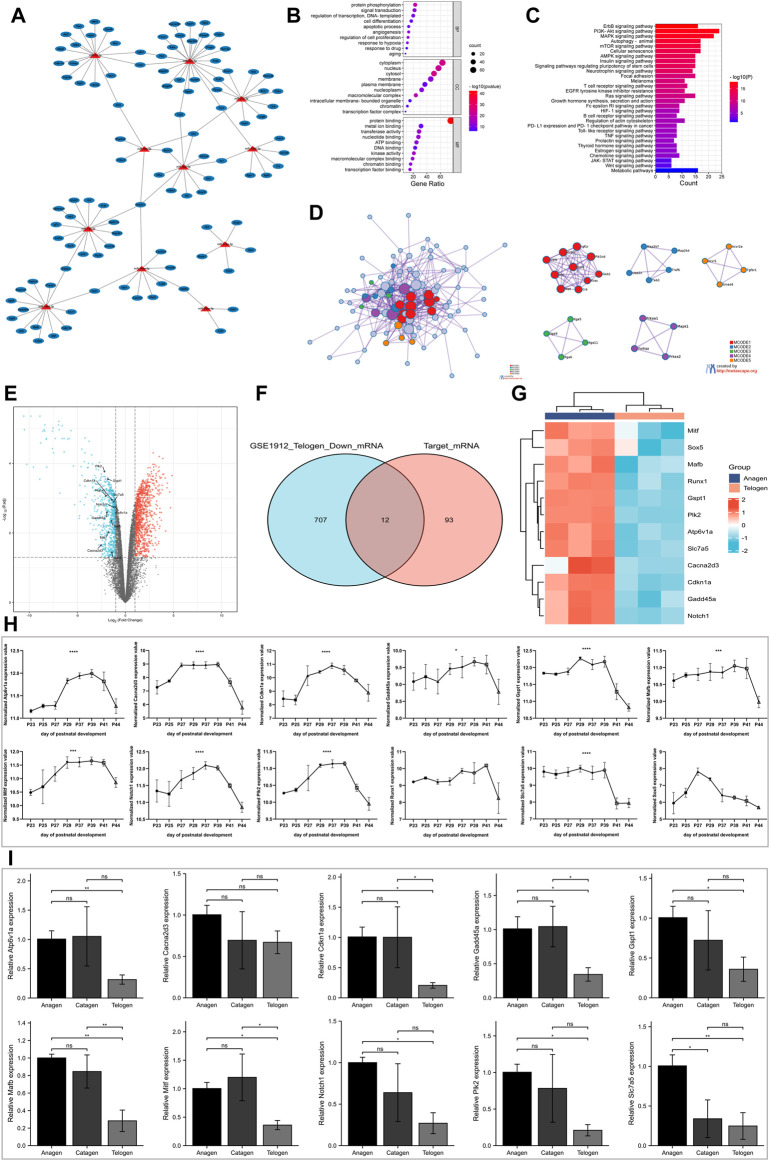
Screening for target mRNA with decreasing expression from the anagen to telogen phase in murine hair follicle cycle. **(A)** Construction of the miRNA-mRNA network. The miRNA-mRNA interactions were experimentally validated in previous studies. **(B)** The GO enrichment analysis of target mRNAs on biological processes, cellular component, and molecular function. **(C)** The KEGG pathway enrichment analysis of target mRNAs. **(D)** The PPI network analysis of target mRNAs. **(E)** Volcano plot shows differentially expressed mRNAs between telogen and anagen phases in the GSE1912 data set. **(F)** The intersection of downregulated mRNAs and target mRNAs. **(G)** Heatmap shows target mRNAs with decreasing expression from the anagen to telogen phase in the GSE1912 data set. **(H)** Validation of identified target mRNAs with a decrease in expression using another data set: GSE11186. The expression of *Atp6v1a*, *Cacna2d3*, *Cdkn1a*, *Gadd45a*, *Gspt1*, *Mafb*, *Mitf*, *Notch1*, *Plk2*, *Runx1*, *Slc7a5*, and *Sox5* at P23-P44 of murine hair follicle cycle. **(I)** Validation of screened target mRNAs using RT-qPCR. The expression of *Atp6v1a*, *Cacna2d3*, *Cdkn1a*, *Gadd45a*, *Gspt1*, *Mafb*, *Mitf*, *Notch1*, *Plk2*, and *Slc7a5* at different stages of murine hair follicle cycle. *, *p* < 0.05; **, *p* < 0.01; ***, *p* < 0.001; ****, *p* < 0.0001. GO, Gene Ontology; KEGG, Kyoto Encyclopedia of Genes and Genomes; PPI, protein–protein interactions; *p*, postnatal.

The interactions between miRNAs and their predicted target mRNAs are available on multiple databases. For our analysis, three databases (TargetScan v8.0, miRDB, and PITA) were used to predict potential miRNA–target mRNA interactions. The mRNAs predicted by all three databases were used for further screening. Furthermore, the miRNA-mRNA interactions that were not experimentally validated in previous studies were excluded. Finally, a list of 105 target mRNAs was obtained, of which 123 miRNA-mRNA pairs were created for our study (Supplementary Table S3). As shown in Figure 3A, a single miRNA can target multiple mRNAs, and several miRNAs in the network can have common target mRNAs.

The DAVID tool was used to perform GO and KEGG pathway enrichment analyses for the 105 target mRNAs. The GO terms “protein phosphorylation,” “angiogenesis,” and “regulation of cell proliferation” were significantly enriched in the BP category. The significantly enriched GO term in the cellular component term was “cytoplasm.” The top three enriched GO terms in the molecular function category were “protein binding,” “metal ion binding,” and “transferase activity” (Figure 3B). The KEGG pathway enrichment analysis revealed the enrichment of several hair growths or development-related pathways, which included the PI3K-Akt signaling, MAPK signaling, and WNT signaling pathways and autophagy (Figure 3C).

Furthermore, the PPI between the target mRNAs was extracted using the Metascape tool to construct the PPI networks. Next, the MCODE algorithm was used to identify densely connected proteins. The GO enrichment analysis was performed on all the MCODE networks (Figure 3D). The results revealed that the genes in mcode 1 were associated with insulin signaling, genes in mcode 2 enriched JNK phosphorylation and activation, genes in mcode 3 were associated with cell adhesion mediated by integrin, genes in mcode 4 were involved in the PI3K-Akt signaling pathway, and genes in mcode 5 participated in positive regulation of pathway-restricted SMAD protein phosphorylation.

The miRNA regulates gene expression by suppressing the translation of mRNA to proteins or by degrading mRNA. Therefore, we investigated the target mRNA whose expression decreased from the anagen phase to the telogen phase. The mRNA expression in the skin of mice at multiple time points was obtained from the GSE1912 data set to identify differentially expressed genes between the telogen and anagen phases. A total of 987 genes were upregulated and 719 genes were downregulated in the skin tissue at the telogen phase when compared to the skin tissue at the anagen phase (Figure 3E). Venn diagram (Figure 3F) showed the intersection of differentially expressed mRNAs that were downregulated and the target mRNAs mentioned previously and was identified as the potential target mRNAs with a decrease in expression from the anagen phase to the telogen phase in the hair follicle cycle. The results revealed 12 target mRNAs with a decrease in expression during the transition. The expression of these mRNA is shown in a heatmap (Figure 3G).

To further validate these results, the skin samples at multiple time points from the GSE11186 data set were analyzed. Figure 3H shows the expression of 12 mRNAs. For murine hair follicle cycle analysis, the hair follicles obtained from the dorsal skin at P23-P25 are likely in the telogen-like stage. At P27-P39, the hair follicles transitioned from an early anagen-like to a late anagen-like stage. At the end of anagen, the hair follicles regressed into the catagen stage (P41) and reentered telogen (P44). The results revealed that the expression values of 10 mRNAs varied periodically, and the expression increased in the anagen phase and decreased in the telogen phase. RT-qPCR was performed to analyze the differential expression of the mRNAs at different stages of the hair follicle cycle. The results revealed a significant decrease in the expression of nine mRNAs (*Atp6v1a*, *Cdkn1a*, *Gadd45a*, *Gspt1*, *Mafb*, *Mitf*, *Notch1*, *Plk2*, and *Slc7a5*) in hair follicles from the anagen phase to the telogen phase (Figure 3I).

### Screening for target lncRNA with decrease in expression from anagen to telogen phases

**FIGURE 4:**
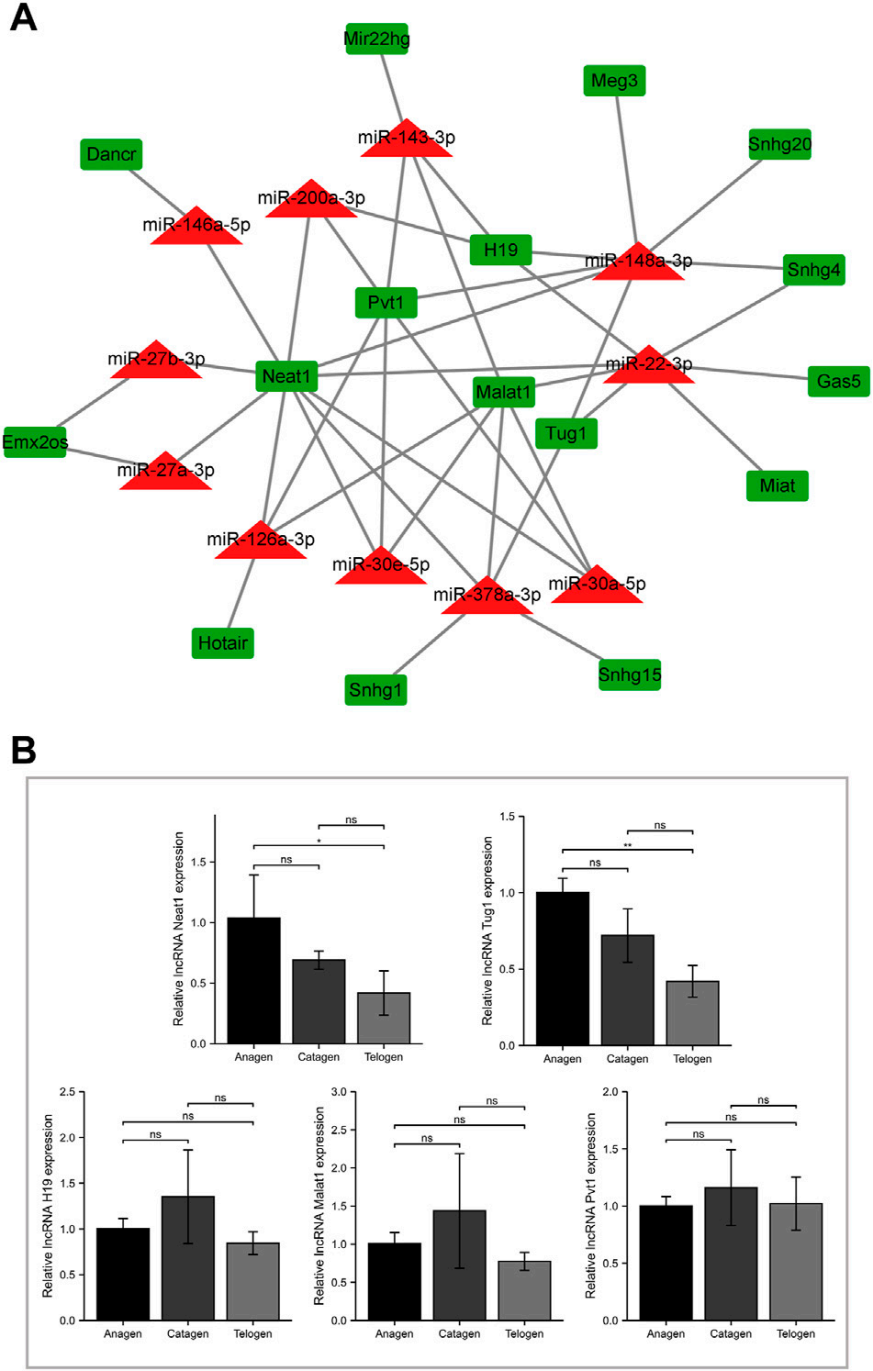
Screening for target lncRNA with decreasing expression from the anagen to telogen phase in murine hair follicle cycle. **(A)** Construction of the miRNA-lncRNA network. The miRNA-lncRNA interactions were experimentally validated in previous studies or predicted using bioinformatic tools. **(B)** Validation of screened lncRNAs using RT-qPCR. The expression of lncRNA H19, Malat1, Neat1, Pvt1, and Tug1 at different stages of murine hair follicle cycle. ns, *p* ≥ 0.05; *, *p* < 0.05; **, *p* < 0.01.

To predict miRNA-lncRNA interactions, the results of the lncRNA subcellular localization, web-based bioinformatic tool ENCORI, and previous literature evidence were integrated based on the ceRNA hypothesis. The results revealed 16 target lncRNAs and 42 miRNA-lncRNA interactions (Figure 4A; Supplementary Table S4). To further screen the target lncRNAs with a decrease in expression from the anagen to telogen phases, the lncRNAs negatively regulated by at least three miRNAs were chosen for validation by RT-qPCR (Figure 4B). Among the five tested lncRNAs, significantly high expressions of lncRNAs Tug1 and Neat1 were observed in the anagen phase, and the expression decreased in the telogen phase. A progressive decrease in the expression trend of target lncRNAs Tug1 and Neat1 from the anagen phase to the telogen phase was observed.

### Construction of competitive endogenous RNA networks of hair follicle cycle and functional enrichment analysis

**FIGURE 5:**
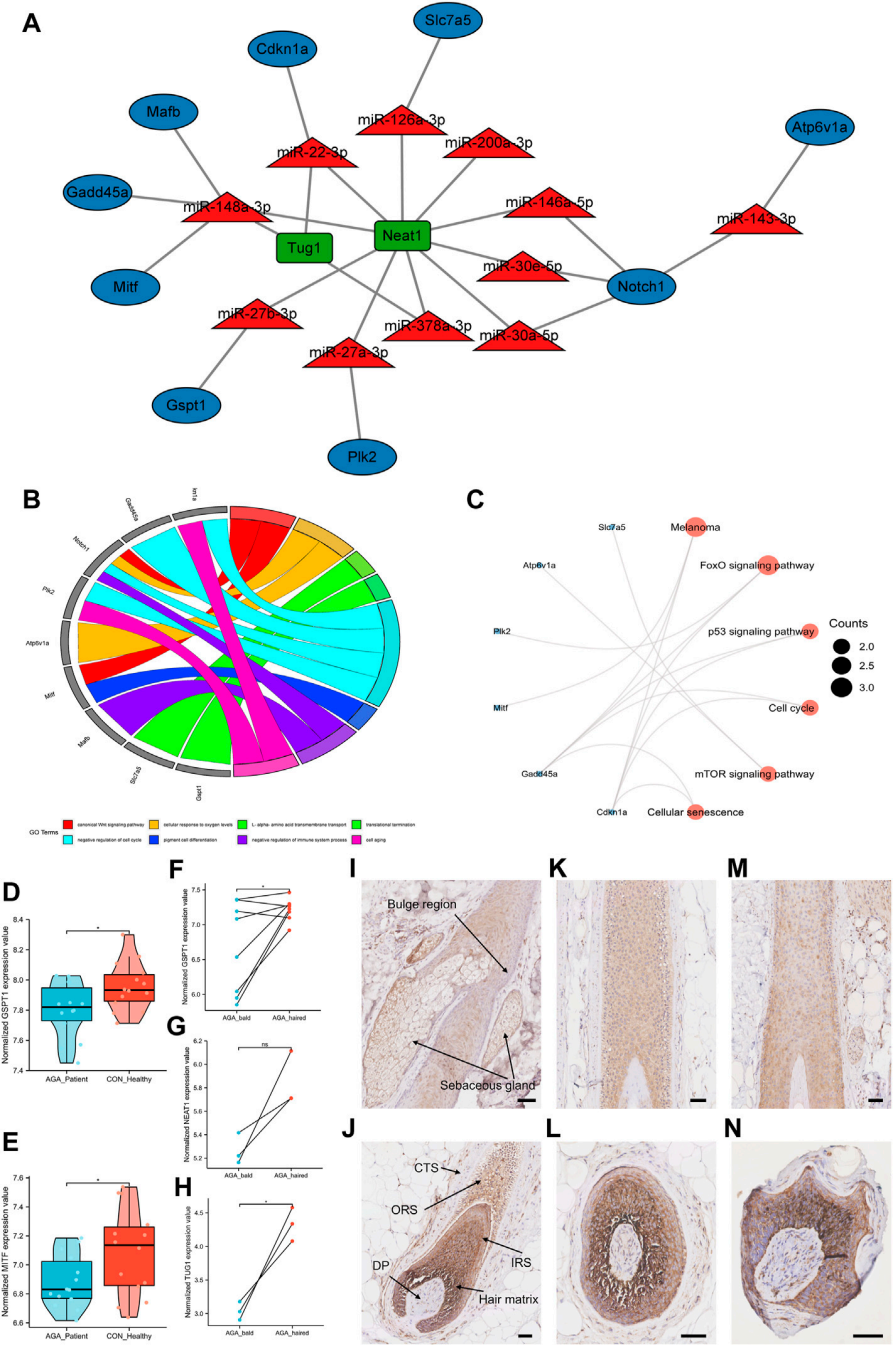
Construction of ceRNA networks of the hair follicle cycle and identification of novel alopecia-related target genes. **(A)** Construction of ceRNA networks in the murine hair follicle cycle based on the identified and PCR-validated genes. **(B)** GO biological processes enrichment analysis of target mRNAs in the ceRNA networks. **(C)** The KEGG pathway enrichment analysis of target mRNAs in the ceRNA networks. **(D,E)** The expression of target genes *GSPT1* and *MITF* in AGA patients and healthy individuals. **(F–H)** The expression of target genes *GSPT1*, lncRNA TUG1, and lncRNA NEAT1 between bald and haired groups in AGA patients. **(I–N)** GSPT1 immunostaining (brown) with blue purple nuclear counterstain in the scalp tissue of healthy individuals **(I–L)** and the bald scalp of patients with AGA **(M,N)**. Anagen follicles of healthy scalp have strong staining for GSPT1 in hair matrix and IRS, medium level in ORS, and low/undetectable staining in the bulge region: DP and CTS. Low GSPT1 expression was observed in upper IRS and ORS of miniaturized hair follicles from the AGA scalp. Scale bars, 50 μm ns, *p* ≥ 0.05; *, *p* < 0.05.

The ceRNA networks in the murine hair follicle cycle were established based on the identified and PCR-validated genes in our study. Eleven miRNAs with an increase in expression, and nine target mRNAs and two target lncRNAs with a decrease in expression during the transition from the anagen to telogen phases were identified in the ceRNA networks (Figure 5A). Finally, we identified six ceRNA networks involved in the hair follicle cycle: Neat1/Tug1-miR-22-3p*-Cdkn1a*, Neat1-miR-27a-3p*-Plk2*, Neat1-miR-27b-3p*-Gspt1*, Neat1-miR-30a/e-5p/miR-146a-5p*-N*otch1, Neat1-miR-126a-3p-Slc7a5, and Neat1/Tug1-miR-148a-3p-Gadd45a/Mafb/Mitf. The GO and KEGG pathway enrichment analyses were performed to explore functional genes from the identified ceRNA networks. GO-BP analysis indicated that Notch1 and Mitf were involved in the WNT signaling pathway. In addition, *Cdkn1a*, *Gadd45a*, *Notch1*, and *Plk2* were enriched in the cell cycle (Figure 5B). The KEGG pathway enrichment analysis revealed that genes associated with *Foxo* and mTOR signaling pathways and cell cycle were enriched (Figure 5C).

### Identification of target genes associated with androgenetic alopecia

To further analyze the ceRNA networks associated with the hair follicle cycle, we explored whether the target genes identified in the ceRNA networks were associated with alopecia. Since there was an increased percentage of telogen hair follicles in patients with AGA, we hypothesized that these target genes would be downregulated in AGA. The data set GSE90594 had scalp samples of 14 AGA patients and 14 healthy individuals. The results revealed that the target genes *GSPT1* and *MITF* were downregulated in AGA patients (Figures 5D,E). Then, data sets (GSE36169 and GSE84839) with paired AGA scalp samples were chosen to compare the expression levels of the target genes between bald and haired scalps. The results revealed that the target mRNA *GSPT1* and lncRNA TUG1 were significantly downregulated in the bald group when compared to the haired group. A decrease in the expression of target lncRNA NEAT1 was observed; however, the difference was not statistically significant (Figures 5F–H). A significantly low *GSPT1* expression was observed in the AGA patients than in the healthy individuals and in the bald scalp of men with AGA than in the adjacent normal tissue (Figures 5D,F). Next, immunohistochemistry was performed to study the localization of GSPT1 protein in human hair follicles from the bald scalp tissue of AGA patients and scalp tissue of healthy individuals. In the scalp tissue of healthy individuals, GSPT1 was primarily expressed in the hair matrix, inner root sheath (IRS), and outer root sheath (ORS). However, no or low GSPT1 expression was observed in the bulge region, dermal papilla (DP), and connective tissue sheath (Figures 5I–L). As shown in Figure 5M, a relatively low GSPT1 expression was observed in the upper IRS and ORS of hair follicles in the AGA patients. Figure 5N shows a miniaturized hair bulb of AGA scalp with high GSPT1 staining in the hair matrix and lighter color intensity in ORS. The immunohistochemistry results are consistent with the results of the microarray analysis.

## Discussion

The incidence of alopecia continues to rise, and the frequency of alopecia increases with age (Adil and Godwin, 2017; Starace et al., 2020). Currently, limited therapies are available for patients with hair loss since the exact mechanisms underlying alopecia and effective therapeutic targets are unavailable (Vasserot et al., 2019; Mirmirani and Fu, 2021). The shorter anagen or longer telogen phases of the hair follicle cycle contribute to the pathogenesis of alopecia. In this study, we analyzed the periodic molecular changes in the hair follicle cycle. Our study focused on the highly expressed miRNAs and their target genes, whose expression levels were low in the telogen phase and high in the anagen phase. To the best of our knowledge, our study is the first to conduct the ceRNA network analysis on the murine hair follicle cycle, and a few novel genes associated with AGA were identified.

Previous studies have shown that an increasing number of miRNAs are associated with hair growth and the hair follicle cycle (Mardaryev et al., 2010; Andl and Botchkareva, 2015). In this study, we reported that 11 miRNAs were upregulated during the catagen phase, peaked in the telogen phase, and were validated using RT-qPCR. Our analysis validated the results of previous studies where the expressions of miR-22, miR-27a/b-3p, mir-30a/e-5p, miR-148a-3p, and miR-200a-3p were upregulated during the telogen phase (Yuan et al., 2015; Bai et al., 2016; Yue et al., 2016; Zhao et al., 2019; Shang et al., 2021). The overexpression of miR-22 in hair follicles led to hair loss by abnormal hair cycling (Yuan et al., 2015). The miR-200 family is involved in cell proliferation and adhesion in hair morphogenesis (Hoefert et al., 2018). miR-143 and miR-148a inhibited DP cell proliferation in the hair follicle of sheep (Lv et al., 2019; Hu et al., 2021). However, previous studies have not reported the functions of miRNAs identified in our study, such as miR-126a-3p, miR-146a-5p, and miR-378a-3p. Based on the miRNA-mRNA interactions supported by experimental evidence from previous studies, we believe that miR-27a/b-3p*-Fzd7*, miR-126a-3p*-Gata3*, and miR-378a-3p*-Grb2* may be involved in the regulation of the hair follicles (Reddy et al., 2004; Mattes et al., 2009; Chen et al., 2013; Genander et al., 2014; Akilli et al., 2015; Geng et al., 2016; Luo et al., 2020). In our study, the expressions of the target genes identified in the ceRNA networks negatively correlated with the screened miRNAs during transition from the anagen to telogen phase. Hence, we hypothesize that mir-30a/e-5p, miR-143-3p, and miR-146a-5p may play a role in the hair follicle cycle by targeting *Notch1*, which plays an important role in hair follicle development and homeostasis (Vauclair et al., 2005). A study reported that in rat hair follicles, high expressions of *Cdkn1a* were found in the anagen phase when compared with the telogen phase (Mitsui et al., 2001). The results are consistent with our results in murine hair follicles. *Mafb* and *Mitf* regulate hair cuticle formation and hair pigmentation, respectively (Hachiya et al., 2009; Miyai et al., 2010). We also speculate that the mir-148a-3p-*Mafb/Mitf* axis may be associated with the maturation of hair shafts during the hair follicle cycle. Our analysis revealed high expressions of five novel genes (*Atp6v1a*, *Gadd45a*, *Gspt1*, *Plk2*, and *Slc7a5*) in the anagen phase which were deregulated in the telogen phase. These genes have never been reported in hair studies previously and require further analysis. Furthermore, lncRNA *Neat1* is associated with skin tumorigenesis (Adriaens et al., 2016). In our study, we predicted that lncRNA *Neat1* sponges 10 miRNAs, which indicates the potential role of lncRNA *Neat1* in cell proliferation of epidermal cells.

The functional enrichment analysis revealed that enriched miRNA was involved in VEGF and VEGFR signaling pathways. A previous study reported that VEGF is important for regulating hair growth and cycle by follicle vascularization (Yano et al., 2001). In our study, the GO enrichment analysis of the 105 target mRNAs revealed enrichment of cell differentiation, proliferation, and apoptosis, but to a lesser extent. During each hair follicle cycle, keratinocytes and fibroblasts of hair follicles undergo sequential proliferation, differentiation, and apoptosis, followed by another wave of proliferation to maintain hair growth (Paus and Cotsarelis, 1999).The KEGG pathway enrichment analysis of the 105 target mRNAs enriched the PI3K-Akt signaling pathway, autophagy, and the WNT signaling pathway, which are involved in the hair follicle cycle. Previous studies have shown that WNT and PI3K-Akt signaling pathways play an important role in hair follicle regeneration and cycle, which is consistent with our results (Huelsken et al., 2001; Lu et al., 2021). In addition, the activation of autophagy can initiate the anagen phase, and impaired autophagy may be associated with alopecia (Chai et al., 2019). We also performed functional enrichment analysis on the nine target mRNAs with decreasing expression from the anagen to telogen phase. GO enrichment analysis confirmed the BP results related to the WNT signaling. The KEGG pathway enrichment analysis revealed the “mTOR signaling pathway,” which regulates autophagy, and the hair cycle was enriched (Kellenberger and Tauchi, 2013; Liu and Sabatini, 2020).

The murine model for synchronized hair follicle cycle aids in the identification of alopecia-related genes in humans (Muller-Rover et al., 2001). Our results have identified that the target genes involved in our ceRNA networks—*GSPT1*, *MITF*, and lncRNA TUG1—were significantly downregulated in AGA patients, which was not reported previously. Interestingly, low expression of *GSPT1* was observed in both AGA patients (compared with healthy individuals) and the bald scalps of AGA patients (compared with the haired scalps of AGA patients). We further confirmed the protein localization of GSPT1 by immunohistochemistry, validating the RNA-profiling results. Previous studies have shown the involvement of *GSPT1* in translation termination and cell proliferation. Furthermore, *GSPT1* silencing suppresses colony formation and cell invasion (Hoshino et al., 1989; Long et al., 2021). Based on the decrease in expression of lncRNA NEAT1 in bald lesions, we hypothesize that the Neat1-miR-27b-3p*-Gspt1* ceRNA network may be involved in AGA progression.

However, our study has a few limitations. First, only a set of miRNAs with increasing expression from anagen to telogen were studied, which formed the basis of subsequent analyses. To comprehensively understand the molecular mechanisms underlying mice hair follicle cycling, further analysis of miRNAs with a decrease in expression and their target genes from the anagen to telogen phase is required. Second, for the analysis, we used publicly available data sets and have not yet performed the next-generation sequencing or array chip analysis on samples collected by us. Hence, additional research using a sensitive and in-depth sequencing method and a larger sample size is required. Third, the number of mice and human scalp samples used for RT-qPCR and immunohistochemistry analysis was small. Hence, a large sample size should be used to validate the results. Fourth, the predicted lncRNA-miRNA-mRNA interactions require further experimental validation, and the underlying mechanism should be investigated. Moreover, the molecular mechanisms associated with the murine hair follicle cycle model may not be directly applicable to humans. Hence, additional experiments are required to validate the significance of genes identified in AGA.

In conclusion, we identified a set of miRNAs with increasing expression during the anagen–telogen phase in the murine hair follicle cycle and explored their functions by target genes enrichment analysis. On further analysis and validation of each component, we successfully constructed potential mRNA-miRNA-lncRNA regulatory networks in the murine hair follicle cycle based on the ceRNA theory. Additionally, our study identified novel genes associated with AGA that are worthy of further analysis.

## Data Availability

The original contributions presented in the study are included in the article/Supplementary material; further inquiries can be directed to the corresponding author.
