# Supplementary material for: An integrative analysis of the lncRNA-miRNA-mRNA competitive endogenous RNA network reveals potential mechanisms in the murine hair follicle cycle
Source: Front Genet. 2022 Oct 25;13:931797. doi: 10.3389/fgene.2022.931797 (PMC9640916; doi:10.3389/fgene.2022.931797)
Supplement: Supplementary file 1 [file Table1.DOCX]

**Table S1. Details of microarray datasets from the GEO database**

| Accession | Platform | Sample_Name | Source_Name | Group |
| --- | --- | --- | --- | --- |
| GSE1912 | GPL81 | GSM34315 | Postnatal day 14, mouse 1 | anagen |
| GSE1912 | GPL81 | GSM34316 | Postnatal day 14, mouse 2 | anagen |
| GSE1912 | GPL81 | GSM34317 | Postnatal day 14, mouse 3 | anagen |
| GSE1912 | GPL81 | GSM34322 | Postnatal day 23, mouse 1 | telogen |
| GSE1912 | GPL81 | GSM34323 | Postnatal day 23, mouse 2 | telogen |
| GSE1912 | GPL81 | GSM34324 | Postnatal day 23, mouse 3 | telogen |
| GSE11186 | GPL1261 | GSM281779 | Postnatal day 23, mouse 1 | telogen |
| GSE11186 | GPL1261 | GSM281780 | Postnatal day 23, mouse 2 | telogen |
| GSE11186 | GPL1261 | GSM281781 | Postnatal day 25, mouse 1 | telogen |
| GSE11186 | GPL1261 | GSM281782 | Postnatal day 25, mouse 2 | telogen |
| GSE11186 | GPL1261 | GSM281783 | Postnatal day 27, mouse 1 | anagen |
| GSE11186 | GPL1261 | GSM281784 | Postnatal day 27, mouse 2 | anagen |
| GSE11186 | GPL1261 | GSM281785 | Postnatal day 27, mouse 3 | anagen |
| GSE11186 | GPL1261 | GSM281786 | Postnatal day 29, mouse 1 | anagen |
| GSE11186 | GPL1261 | GSM281787 | Postnatal day 29, mouse 2 | anagen |
| GSE11186 | GPL1261 | GSM281788 | Postnatal day 29, mouse 3 | anagen |
| GSE11186 | GPL1261 | GSM281790 | Postnatal day 37, mouse 1 | anagen |
| GSE11186 | GPL1261 | GSM281791 | Postnatal day 37, mouse 2 | anagen |
| GSE11186 | GPL1261 | GSM281792 | Postnatal day 37, mouse 3 | anagen |
| GSE11186 | GPL1261 | GSM281793 | Postnatal day 39, mouse 1 | anagen |
| GSE11186 | GPL1261 | GSM281794 | Postnatal day 39, mouse 2 | anagen |
| GSE11186 | GPL1261 | GSM281795 | Postnatal day 39, mouse 3 | anagen |
| GSE11186 | GPL1261 | GSM281796 | Postnatal day 41, mouse 1 | catagen |
| GSE11186 | GPL1261 | GSM281797 | Postnatal day 41, mouse 2 | catagen |
| GSE11186 | GPL1261 | GSM281798 | Postnatal day 41, mouse 3 | catagen |
| GSE11186 | GPL1261 | GSM281799 | Postnatal day 44, mouse 1 | telogen |
| GSE11186 | GPL1261 | GSM281800 | Postnatal day 44, mouse 2 | telogen |
| GSE11186 | GPL1261 | GSM281801 | Postnatal day 44, mouse 3 | telogen |
| GSE90594 | GPL17077 | GSM2407372 | androgenetic alopecia patient 1 | AGA_patient |
| GSE90594 | GPL17077 | GSM2407373 | androgenetic alopecia patient 2 | AGA_patient |
| GSE90594 | GPL17077 | GSM2407374 | androgenetic alopecia patient 3 | AGA_patient |
| GSE90594 | GPL17077 | GSM2407375 | androgenetic alopecia patient 4 | AGA_patient |
| GSE90594 | GPL17077 | GSM2407376 | androgenetic alopecia patient 5 | AGA_patient |
| GSE90594 | GPL17077 | GSM2407377 | androgenetic alopecia patient 6 | AGA_patient |
| GSE90594 | GPL17077 | GSM2407378 | androgenetic alopecia patient 7 | AGA_patient |
| GSE90594 | GPL17077 | GSM2407379 | androgenetic alopecia patient 8 | AGA_patient |
| GSE90594 | GPL17077 | GSM2407380 | androgenetic alopecia patient 9 | AGA_patient |
| GSE90594 | GPL17077 | GSM2407381 | androgenetic alopecia patient 10 | AGA_patient |
| GSE90594 | GPL17077 | GSM2407382 | androgenetic alopecia patient 11 | AGA_patient |
| GSE90594 | GPL17077 | GSM2407383 | androgenetic alopecia patient 12 | AGA_patient |
| GSE90594 | GPL17077 | GSM2407384 | androgenetic alopecia patient 13 | AGA_patient |
| GSE90594 | GPL17077 | GSM2407385 | androgenetic alopecia patient 14 | AGA_patient |
| GSE90594 | GPL17077 | GSM2407386 | healthy control 1 | CON_healthy |
| GSE90594 | GPL17077 | GSM2407387 | healthy control 2 | CON_healthy |
| GSE90594 | GPL17077 | GSM2407388 | healthy control 3 | CON_healthy |
| GSE90594 | GPL17077 | GSM2407389 | healthy control 4 | CON_healthy |
| GSE90594 | GPL17077 | GSM2407390 | healthy control 5 | CON_healthy |
| GSE90594 | GPL17077 | GSM2407391 | healthy control 6 | CON_healthy |
| GSE90594 | GPL17077 | GSM2407392 | healthy control 7 | CON_healthy |
| GSE90594 | GPL17077 | GSM2407393 | healthy control 8 | CON_healthy |
| GSE90594 | GPL17077 | GSM2407394 | healthy control 9 | CON_healthy |
| GSE90594 | GPL17077 | GSM2407395 | healthy control 10 | CON_healthy |
| GSE90594 | GPL17077 | GSM2407396 | healthy control 11 | CON_healthy |
| GSE90594 | GPL17077 | GSM2407397 | healthy control 12 | CON_healthy |
| GSE90594 | GPL17077 | GSM2407398 | healthy control 13 | CON_healthy |
| GSE90594 | GPL17077 | GSM2407399 | healthy control 14 | CON_healthy |
| GSE36169 | GPL96 | GSM882149 | androgenetic alopecia patient 1-Haired | AGA_haired |
| GSE36169 | GPL96 | GSM882150 | androgenetic alopecia patient 1-Bald | AGA_bald |
| GSE36169 | GPL96 | GSM882151 | androgenetic alopecia patient 2-Haired | AGA_haired |
| GSE36169 | GPL96 | GSM882152 | androgenetic alopecia patient 2-Bald | AGA_bald |
| GSE36169 | GPL96 | GSM882153 | androgenetic alopecia patient 3-Haired | AGA_haired |
| GSE36169 | GPL96 | GSM882154 | androgenetic alopecia patient 3-Bald | AGA_bald |
| GSE36169 | GPL96 | GSM882155 | androgenetic alopecia patient 4-Haired | AGA_haired |
| GSE36169 | GPL96 | GSM882156 | androgenetic alopecia patient 4-Bald | AGA_bald |
| GSE36169 | GPL96 | GSM882157 | androgenetic alopecia patient 5-Haired | AGA_haired |
| GSE36169 | GPL96 | GSM882158 | androgenetic alopecia patient 5-Bald | AGA_bald |
| GSE84839 | GPL21827 | GSM2252085 | androgenetic alopecia patient 1-Bald | AGA_bald |
| GSE84839 | GPL21827 | GSM2252086 | androgenetic alopecia patient 1-Haired | AGA_haired |
| GSE84839 | GPL21827 | GSM2252087 | androgenetic alopecia patient 2-Bald | AGA_bald |
| GSE84839 | GPL21827 | GSM2252088 | androgenetic alopecia patient 2-Haired | AGA_haired |
| GSE84839 | GPL21827 | GSM2252089 | androgenetic alopecia patient 3-Bald | AGA_bald |
| GSE84839 | GPL21827 | GSM2252090 | androgenetic alopecia patient 3-Haired | AGA_haired |

Abbreviations: AGA: androgenetic alopecia; CON: control
