# Supplementary material for: An integrative analysis of the lncRNA-miRNA-mRNA competitive endogenous RNA network reveals potential mechanisms in the murine hair follicle cycle
Source: Front Genet. 2022 Oct 25;13:931797. doi: 10.3389/fgene.2022.931797 (PMC9640916; doi:10.3389/fgene.2022.931797)
Supplement: Supplementary file 2 [file Table2.DOCX]

**Table S2. Sequences of primers used for RT-qPCR**

| Primer Name | Forward sequence (5′-3′) | Reverse sequence (5′-3′) |
| --- | --- | --- |
| Atp6v1a | GCTGGCTTCTTTCTATGAGCGAG | GCGTTGCAGAAGTGACTGGATC |
| Cacna2d3 | GAGGCTGCTTTTGCCGACAATC | TAGGTCGGCTAAGCACATGGAG |
| Cdkn1a | TCGCTGTCTTGCACTCTGGTGT | CCAATCTGCGCTTGGAGTGATAG |
| Gadd45a | CCTGGAGGAAGTGCTCAGCAAG | GTCGTCTTCGTCAGCAGCCAG |
| Gspt1 | CGGTAAACTGGAGCAACGAGAG | TGTCAGTCCTGAGCACGGCATA |
| Mafb | TACTGGATGGCGAGCAACTACC | ACTACGGAAGCCGTCGAAGCTC |
| Mitf | GATCGACCTCTACAGCAACCAG | GCTCTTGCTTCAGACTCTGTGG |
| Notch1 | GCTGCCTCTTTGATGGCTTCGA | CACATTCGGCACTGTTACAGCC |
| Plk2 | GGCAGATTGTGTCAGGACTCAAG | TGGTTCCAGTCTGGCTGCCAAA |
| Slc7a5 | GGTCTCTGTTCACGTCCTCAAG | GAACACCAGTGATGGCACAGGT |
| Mouse H19 | GCACTAAGTCGATTGCACTGG | GCCTCAAGCACACGGCCACA |
| Malat1 | GGACTTGAGCTGAGGTGCTT | GCTTCACCACCACATCCGTA |
| Neat1 | TGGAGATTGAAGGCGCAAGT | ACCACAGAAGAGGAAGCACG |
| Tug1 | CATCTCACAAGGCTTCAACCA | ACCTCAACTCCCACTTCACTA |
| Pvt1 | CGGAGGCAATCCTATAAGACA | GCTGGATCTATCACCTGAGCA |
| Gapdh | CATCACTGCCACCCAGAAGACTG | ATGCCAGTGAGCTTCCCGTTCAG |
| miR-148a-3p | TCAGTGCACTACAGAACTTTGT | the mRQ 3’ Primer supplied with the kit |
| miR-146a-5p | TGAGAACTGAATTCCATGGGTT | the mRQ 3’ Primer supplied with the kit |
| miR-200a-3p | TAACACTGTCTGGTAACGATGT | the mRQ 3’ Primer supplied with the kit |
| miR-30e-5p | TGTAAACATCCTTGACTGGAAG | the mRQ 3’ Primer supplied with the kit |
| miR-30a-5p | TGTAAACATCCTCGACTGGAAG | the mRQ 3’ Primer supplied with the kit |
| miR-27a-3p | TTCACAGTGGCTAAGTTCCGC | the mRQ 3’ Primer supplied with the kit |
| miR-143-3p | TGAGATGAAGCACTGTAGCTC | the mRQ 3’ Primer supplied with the kit |
| miR-27b-3p | TTCACAGTGGCTAAGTTCTGC | the mRQ 3’ Primer supplied with the kit |
| miR-126a-3p | TCGTACCGTGAGTAATAATGCG | the mRQ 3’ Primer supplied with the kit |
| miR-378a-3p | ACTGGACTTGGAGTCAGAAGG | the mRQ 3’ Primer supplied with the kit |
| miR-22-3p | AAGCTGCCAGTTGAAGAACTGT | the mRQ 3’ Primer supplied with the kit |
| U6 | U6 Forward Primer supplied with the kit | U6 Reverse Primer supplied with the kit |
