# Supplementary material for: An integrative analysis of the lncRNA-miRNA-mRNA competitive endogenous RNA network reveals potential mechanisms in the murine hair follicle cycle
Source: Front Genet. 2022 Oct 25;13:931797. doi: 10.3389/fgene.2022.931797 (PMC9640916; doi:10.3389/fgene.2022.931797)
Supplement: Supplementary file 3 [file Table3.DOCX]

**Table S3. Identification of miRNA–mRNA interactions with experimental evidence**

| miRNA | Target_mRNA | Reference_PMID | |
| --- | --- | --- | --- |
| miR-22-3p | Akt3 | [25323119](https://www.ncbi.nlm.nih.gov/pubmed/25323119) | [32572913](https://www.ncbi.nlm.nih.gov/pubmed/32572913) |
| miR-22-3p | Cdkn1a | [21565979](https://www.ncbi.nlm.nih.gov/pubmed/21565979) |  |
| miR-22-3p | Creb1 | [32300145](https://www.ncbi.nlm.nih.gov/pubmed/32300145) |  |
| miR-22-3p | Ddit4 | [32151711](https://www.ncbi.nlm.nih.gov/pubmed/32151711) |  |
| miR-22-3p | Elovl6 | [28445717](https://www.ncbi.nlm.nih.gov/pubmed/28445717) |  |
| miR-22-3p | Hipk1 | [28987030](https://www.ncbi.nlm.nih.gov/pubmed/28987030) |  |
| miR-22-3p | Jarid2 | [34094675](https://www.ncbi.nlm.nih.gov/pubmed/34094675) |  |
| miR-22-3p | Max | [20214878](https://www.ncbi.nlm.nih.gov/pubmed/20214878) |  |
| miR-22-3p | Mthfr | [33168819](https://www.ncbi.nlm.nih.gov/pubmed/33168819) |  |
| miR-22-3p | Pten | [21618527](https://www.ncbi.nlm.nih.gov/pubmed/21618527) | [29850604](https://www.ncbi.nlm.nih.gov/pubmed/29850604) |
| miR-22-3p | Rab5b | [27569217](https://www.ncbi.nlm.nih.gov/pubmed/27569217) |  |
| miR-22-3p | Rgs2 | [21168126](https://www.ncbi.nlm.nih.gov/pubmed/21168126) |  |
| miR-22-3p | Sirt1 | [35167638](https://www.ncbi.nlm.nih.gov/pubmed/35167638) |  |
| miR-22-3p | Stag2 | [28161397](https://www.ncbi.nlm.nih.gov/pubmed/28161397) |  |
| miR-22-3p | Tgfbr1 | [29588073](https://www.ncbi.nlm.nih.gov/pubmed/29588073) | [27997889](https://www.ncbi.nlm.nih.gov/pubmed/27997889) |
| miR-22-3p | Tiam1 | [25926441](https://www.ncbi.nlm.nih.gov/pubmed/25926441) |  |
| miR-22-3p | Ywhaz | [23355742](https://www.ncbi.nlm.nih.gov/pubmed/23355742) |  |
| miR-27a-3p | Acvr2a | [25369332](https://www.ncbi.nlm.nih.gov/pubmed/25369332) |  |
| miR-27a-3p | Apaf1 | [32190895](https://www.ncbi.nlm.nih.gov/pubmed/32190895) | [31452277](https://www.ncbi.nlm.nih.gov/pubmed/31452277) |
| miR-27a-3p | B4galt3 | [26987623](https://www.ncbi.nlm.nih.gov/pubmed/26987623) |  |
| miR-27a-3p | Cacna2d3 | [30327467](https://www.ncbi.nlm.nih.gov/pubmed/30327467) |  |
| miR-27a-3p | Creb1 | [34350751](https://www.ncbi.nlm.nih.gov/pubmed/34350751) | [24903074](https://www.ncbi.nlm.nih.gov/pubmed/24903074) |
| miR-27a-3p | Fosb | [32819575](https://www.ncbi.nlm.nih.gov/pubmed/32819575) |  |
| miR-27a-3p | Foxo1 | [33875617](https://www.ncbi.nlm.nih.gov/pubmed/33875617) |  |
| miR-27a-3p | Fzd7 | [24018051](https://www.ncbi.nlm.nih.gov/pubmed/24018051) |  |
| miR-27a-3p | Gfpt2 | [33910455](https://www.ncbi.nlm.nih.gov/pubmed/33910455) |  |
| miR-27a-3p | Hmgcr | [32975270](https://www.ncbi.nlm.nih.gov/pubmed/32975270) |  |
| miR-27a-3p | Map2k4 | [34219538](https://www.ncbi.nlm.nih.gov/pubmed/34219538) | [27594411](https://www.ncbi.nlm.nih.gov/pubmed/27594411) |
| miR-27a-3p | Map2k7 | [23034448](https://www.ncbi.nlm.nih.gov/pubmed/23034448) |  |
| miR-27a-3p | Nedd4 | [33262943](https://www.ncbi.nlm.nih.gov/pubmed/33262943) |  |
| miR-27a-3p | Nf1 | [33570696](https://www.ncbi.nlm.nih.gov/pubmed/33570696) |  |
| miR-27a-3p | Pdpk1 | [33515696](https://www.ncbi.nlm.nih.gov/pubmed/33515696) |  |
| miR-27a-3p | Plk2 | [31134620](https://www.ncbi.nlm.nih.gov/pubmed/31134620) |  |
| miR-27a-3p | Pparg | [31554889](https://www.ncbi.nlm.nih.gov/pubmed/31554889) |  |
| miR-27a-3p | Prkaa2 | [29462799](https://www.ncbi.nlm.nih.gov/pubmed/29462799) |  |
| miR-27a-3p | Rara | [25915942](https://www.ncbi.nlm.nih.gov/pubmed/25915942) |  |
| miR-27a-3p | Runx1 | [19298589](https://www.ncbi.nlm.nih.gov/pubmed/19298589) |  |
| miR-27a-3p | Sgpp1 | [25166914](https://www.ncbi.nlm.nih.gov/pubmed/25166914) |  |
| miR-27a-3p | Tab3 | [29156532](https://www.ncbi.nlm.nih.gov/pubmed/29156532) | [23034448](https://www.ncbi.nlm.nih.gov/pubmed/23034448) |
| miR-27a-3p | Tgfbr1 | [29531222](https://www.ncbi.nlm.nih.gov/pubmed/29531222) |  |
| miR-27a-3p | Tsc1 | [32410883](https://www.ncbi.nlm.nih.gov/pubmed/32410883) |  |
| miR-27a-3p | Ube2n | [34160887](https://www.ncbi.nlm.nih.gov/pubmed/34160887) |  |
| miR-27b-3p | Adora2b | [30429458](https://www.ncbi.nlm.nih.gov/pubmed/30429458) |  |
| miR-27b-3p | Bmi1 | [27716060](https://www.ncbi.nlm.nih.gov/pubmed/27716060) |  |
| miR-27b-3p | Cblb | [29416005](https://www.ncbi.nlm.nih.gov/pubmed/29416005) |  |
| miR-27b-3p | Dll4 | [22207734](https://www.ncbi.nlm.nih.gov/pubmed/22207734) |  |
| miR-27b-3p | Fzd7 | [26780940](https://www.ncbi.nlm.nih.gov/pubmed/26780940) |  |
| miR-27b-3p | Gspt1 | [31539861](https://www.ncbi.nlm.nih.gov/pubmed/31539861) |  |
| miR-27b-3p | Met | [29135973](https://www.ncbi.nlm.nih.gov/pubmed/29135973) |  |
| miR-27b-3p | Pdhx | [30012170](https://www.ncbi.nlm.nih.gov/pubmed/30012170) |  |
| miR-27b-3p | Pparg | [32850439](https://www.ncbi.nlm.nih.gov/pubmed/32850439) |  |
| miR-27b-3p | Runx1 | [34238170](https://www.ncbi.nlm.nih.gov/pubmed/34238170) |  |
| miR-27b-3p | Vegfb | [30328349](https://www.ncbi.nlm.nih.gov/pubmed/30328349) |  |
| miR-30a-5p | Becn1 | [34080645](https://www.ncbi.nlm.nih.gov/pubmed/34080645) | [33841675](https://www.ncbi.nlm.nih.gov/pubmed/33841675) |
| miR-30a-5p | Bnip3l | [35269458](https://www.ncbi.nlm.nih.gov/pubmed/35269458) |  |
| miR-30a-5p | Dll4 | [23817492](https://www.ncbi.nlm.nih.gov/pubmed/23817492) | [23826258](https://www.ncbi.nlm.nih.gov/pubmed/23826258) |
| miR-30a-5p | Irs1 | [30723145](https://www.ncbi.nlm.nih.gov/pubmed/30723145) |  |
| miR-30a-5p | Me1 | [28475173](https://www.ncbi.nlm.nih.gov/pubmed/28475173) |  |
| miR-30a-5p | Nfatc3 | [26473838](https://www.ncbi.nlm.nih.gov/pubmed/26473838) | [26436650](https://www.ncbi.nlm.nih.gov/pubmed/26436650) |
| miR-30a-5p | Notch1 | [24029422](https://www.ncbi.nlm.nih.gov/pubmed/24029422) |  |
| miR-30a-5p | Nt5e | [32067282](https://www.ncbi.nlm.nih.gov/pubmed/32067282) |  |
| miR-30a-5p | Pik3cd | [23486085](https://www.ncbi.nlm.nih.gov/pubmed/23486085) |  |
| miR-30a-5p | Skil | [30279543](https://www.ncbi.nlm.nih.gov/pubmed/30279543) |  |
| miR-30a-5p | Tab3 | [26555189](https://www.ncbi.nlm.nih.gov/pubmed/26555189) |  |
| miR-30e-5p | Becn1 | [34042027](https://www.ncbi.nlm.nih.gov/pubmed/34042027) |  |
| miR-30e-5p | Dll4 | [31134094](https://www.ncbi.nlm.nih.gov/pubmed/31134094) |  |
| miR-30e-5p | Notch1 | [29899524](https://www.ncbi.nlm.nih.gov/pubmed/29899524) |  |
| miR-30e-5p | P4ha1 | [26966067](https://www.ncbi.nlm.nih.gov/pubmed/26966067) |  |
| miR-30e-5p | Ppp3cb | [26436650](https://www.ncbi.nlm.nih.gov/pubmed/26436650) |  |
| miR-30e-5p | Sirt1 | [29174979](https://www.ncbi.nlm.nih.gov/pubmed/29174979) |  |
| miR-126a-3p | Crk | [25027343](https://www.ncbi.nlm.nih.gov/pubmed/25027343) | [24969300](https://www.ncbi.nlm.nih.gov/pubmed/24969300) |
| miR-126a-3p | Gata3 | [19843690](https://www.ncbi.nlm.nih.gov/pubmed/19843690) |  |
| miR-126a-3p | Irs1 | [33501603](https://www.ncbi.nlm.nih.gov/pubmed/33501603) | [28987000](https://www.ncbi.nlm.nih.gov/pubmed/28987000) |
| miR-126a-3p | Itga11 | [26194885](https://www.ncbi.nlm.nih.gov/pubmed/26194885) |  |
| miR-126a-3p | Itga6 | [33317527](https://www.ncbi.nlm.nih.gov/pubmed/33317527) |  |
| miR-126a-3p | Slc7a5 | [32212853](https://www.ncbi.nlm.nih.gov/pubmed/32212853) |  |
| miR-126a-3p | Tsc1 | [30341633](https://www.ncbi.nlm.nih.gov/pubmed/30341633) |  |
| miR-143-3p | Abl2 | [32196963](https://www.ncbi.nlm.nih.gov/pubmed/32196963) |  |
| miR-143-3p | Atg2b | [28391715](https://www.ncbi.nlm.nih.gov/pubmed/28391715) | [29562274](https://www.ncbi.nlm.nih.gov/pubmed/29562274) |
| miR-143-3p | Atp6v1a | [29274513](https://www.ncbi.nlm.nih.gov/pubmed/29274513) | [26589421](https://www.ncbi.nlm.nih.gov/pubmed/26589421) |
| miR-143-3p | Dnmt3a | [19638978](https://www.ncbi.nlm.nih.gov/pubmed/19638978) | [28619512](https://www.ncbi.nlm.nih.gov/pubmed/28619512) |
| miR-143-3p | Gabarapl1 | [26349981](https://www.ncbi.nlm.nih.gov/pubmed/26349981) |  |
| miR-143-3p | Hk2 | [32006291](https://www.ncbi.nlm.nih.gov/pubmed/32006291) | [30825877](https://www.ncbi.nlm.nih.gov/pubmed/30825877) |
| miR-143-3p | Igf1r | [31443963](https://www.ncbi.nlm.nih.gov/pubmed/31443963) | [25474488](https://www.ncbi.nlm.nih.gov/pubmed/25474488) |
| miR-143-3p | Kat6a | [34459452](https://www.ncbi.nlm.nih.gov/pubmed/34459452) |  |
| miR-143-3p | Kras | [31066120](https://www.ncbi.nlm.nih.gov/pubmed/31066120) | [31521891](https://www.ncbi.nlm.nih.gov/pubmed/31521891) |
| miR-143-3p | Limk1 | [25003638](https://www.ncbi.nlm.nih.gov/pubmed/25003638) | [28559978](https://www.ncbi.nlm.nih.gov/pubmed/28559978) |
| miR-143-3p | Map3k7 | [32647147](https://www.ncbi.nlm.nih.gov/pubmed/32647147) | [28746466](https://www.ncbi.nlm.nih.gov/pubmed/28746466) |
| miR-143-3p | Mybl2 | [29268817](https://www.ncbi.nlm.nih.gov/pubmed/29268817) |  |
| miR-143-3p | Notch1 | [21685392](https://www.ncbi.nlm.nih.gov/pubmed/21685392) |  |
| miR-143-3p | Ralbp1 | [31336396](https://www.ncbi.nlm.nih.gov/pubmed/31336396) |  |
| miR-143-3p | Sox5 | [33824420](https://www.ncbi.nlm.nih.gov/pubmed/33824420) |  |
| miR-146a-5p | Camk2d | [28383548](https://www.ncbi.nlm.nih.gov/pubmed/28383548) |  |
| miR-146a-5p | Card10 | [22992343](https://www.ncbi.nlm.nih.gov/pubmed/22992343) |  |
| miR-146a-5p | Irak1 | [29544526](https://www.ncbi.nlm.nih.gov/pubmed/29544526) | [34733985](https://www.ncbi.nlm.nih.gov/pubmed/34733985) |
| miR-146a-5p | Med1 | [31894315](https://www.ncbi.nlm.nih.gov/pubmed/31894315) |  |
| miR-146a-5p | Notch1 | [29575548](https://www.ncbi.nlm.nih.gov/pubmed/29575548) | [30103197](https://www.ncbi.nlm.nih.gov/pubmed/30103197) |
| miR-146a-5p | Smad4 | [34868004](https://www.ncbi.nlm.nih.gov/pubmed/34868004) | [28510617](https://www.ncbi.nlm.nih.gov/pubmed/28510617) |
| miR-146a-5p | Sos1 | [25469565](https://www.ncbi.nlm.nih.gov/pubmed/25469565) |  |
| miR-146a-5p | Stat5b | [33162030](https://www.ncbi.nlm.nih.gov/pubmed/33162030) | [22529366](https://www.ncbi.nlm.nih.gov/pubmed/22529366) |
| miR-146a-5p | Traf6 | [34733985](https://www.ncbi.nlm.nih.gov/pubmed/34733985) | [33897883](https://www.ncbi.nlm.nih.gov/pubmed/33897883) |
| miR-148a-3p | Acvr1 | [32904700](https://www.ncbi.nlm.nih.gov/pubmed/32904700) | [25271001](https://www.ncbi.nlm.nih.gov/pubmed/25271001) |
| miR-148a-3p | Cckbr | [23975374](https://www.ncbi.nlm.nih.gov/pubmed/23975374) | [21168126](https://www.ncbi.nlm.nih.gov/pubmed/21168126) |
| miR-148a-3p | Cdkn1b | [27842905](https://www.ncbi.nlm.nih.gov/pubmed/27842905) |  |
| miR-148a-3p | Gadd45a | [26901150](https://www.ncbi.nlm.nih.gov/pubmed/26901150) |  |
| miR-148a-3p | Itga5 | [34135940](https://www.ncbi.nlm.nih.gov/pubmed/34135940) |  |
| miR-148a-3p | Itga9 | [31489579](https://www.ncbi.nlm.nih.gov/pubmed/31489579) |  |
| miR-148a-3p | Klf4 | [31104329](https://www.ncbi.nlm.nih.gov/pubmed/31104329) |  |
| miR-148a-3p | Mafb | [32213710](https://www.ncbi.nlm.nih.gov/pubmed/32213710) |  |
| miR-148a-3p | Mitf | [20644734](https://www.ncbi.nlm.nih.gov/pubmed/20644734) |  |
| miR-148a-3p | Prkaa1 | [30201950](https://www.ncbi.nlm.nih.gov/pubmed/30201950) |  |
| miR-148a-3p | Rab14 | [28098870](https://www.ncbi.nlm.nih.gov/pubmed/28098870) |  |
| miR-148a-3p | Ralbp1 | [27748916](https://www.ncbi.nlm.nih.gov/pubmed/27748916) |  |
| miR-148a-3p | Serpine1 | [35218720](https://www.ncbi.nlm.nih.gov/pubmed/35218720) |  |
| miR-148a-3p | Sos1 | [28915601](https://www.ncbi.nlm.nih.gov/pubmed/28915601) |  |
| miR-200a-3p | E2f3 | [34634218](https://www.ncbi.nlm.nih.gov/pubmed/34634218) | [32684096](https://www.ncbi.nlm.nih.gov/pubmed/32684096) |
| miR-200a-3p | Gab1 | [28081727](https://www.ncbi.nlm.nih.gov/pubmed/28081727) |  |
| miR-200a-3p | Stat5b | [34716993](https://www.ncbi.nlm.nih.gov/pubmed/34716993) |  |
| miR-378a-3p | Dusp1 | [32463795](https://www.ncbi.nlm.nih.gov/pubmed/32463795) |  |
| miR-378a-3p | Gli3 | [27001906](https://www.ncbi.nlm.nih.gov/pubmed/27001906) | [29915286](https://www.ncbi.nlm.nih.gov/pubmed/29915286) |
| miR-378a-3p | Grb2 | [32605923](https://www.ncbi.nlm.nih.gov/pubmed/32605923) | [31220469](https://www.ncbi.nlm.nih.gov/pubmed/31220469) |
| miR-378a-3p | Mapk1 | [23625957](https://www.ncbi.nlm.nih.gov/pubmed/23625957) | [28053239](https://www.ncbi.nlm.nih.gov/pubmed/28053239) |
| miR-378a-3p | Npnt | [28476557](https://www.ncbi.nlm.nih.gov/pubmed/28476557) |  |
