# Supplementary material for: An integrative analysis of the lncRNA-miRNA-mRNA competitive endogenous RNA network reveals potential mechanisms in the murine hair follicle cycle
Source: Front Genet. 2022 Oct 25;13:931797. doi: 10.3389/fgene.2022.931797 (PMC9640916; doi:10.3389/fgene.2022.931797)
Supplement: Supplementary file 4 [file Table4.DOCX]

**T****able S4. Identification of miRNA–lncRNA interactions with experimental or predictive evidence**

| miRNA | Target_lncRNA | Reference_PMID | |
| --- | --- | --- | --- |
| miR-22-3p | Gas5 | predicted |  |
| miR-22-3p | H19 | [33267897](https://www.ncbi.nlm.nih.gov/pubmed/33267897) | [31755219](https://www.ncbi.nlm.nih.gov/pubmed/31755219) |
| miR-22-3p | Malat1 | [33135336](https://www.ncbi.nlm.nih.gov/pubmed/33135336) | [32104001](https://www.ncbi.nlm.nih.gov/pubmed/32104001) |
| miR-22-3p | Miat | [28703801](https://www.ncbi.nlm.nih.gov/pubmed/28703801) |  |
| miR-22-3p | Neat1 | [34081624](https://www.ncbi.nlm.nih.gov/pubmed/34081624) | [33839999](https://www.ncbi.nlm.nih.gov/pubmed/33839999) |
| miR-22-3p | Snhg4 | predicted |  |
| miR-22-3p | Tug1 | predicted |  |
| miR-27a-3p | Emx2os | predicted |  |
| miR-27a-3p | Neat1 | [34239033](https://www.ncbi.nlm.nih.gov/pubmed/34239033) | [32606599](https://www.ncbi.nlm.nih.gov/pubmed/32606599) |
| miR-27b-3p | Emx2os | predicted |  |
| miR-27b-3p | Neat1 | [34497381](https://www.ncbi.nlm.nih.gov/pubmed/34497381) | [33292252](https://www.ncbi.nlm.nih.gov/pubmed/33292252) |
| miR-30a-5p | Malat1 | [33192306](https://www.ncbi.nlm.nih.gov/pubmed/33192306) | [33192306](https://www.ncbi.nlm.nih.gov/pubmed/33192306) |
| miR-30a-5p | Neat1 | [34396632](https://www.ncbi.nlm.nih.gov/pubmed/34396632) |  |
| miR-30a-5p | Pvt1 | [33042437](https://www.ncbi.nlm.nih.gov/pubmed/33042437) | [32557622](https://www.ncbi.nlm.nih.gov/pubmed/32557622) |
| miR-30e-5p | Malat1 | [33192306](https://www.ncbi.nlm.nih.gov/pubmed/33192306) | [33192306](https://www.ncbi.nlm.nih.gov/pubmed/33192306) |
| miR-30e-5p | Neat1 | [35203705](https://www.ncbi.nlm.nih.gov/pubmed/35203705) |  |
| miR-30e-5p | Pvt1 | predicted |  |
| miR-126a-3p | Hotair | [32964995](https://www.ncbi.nlm.nih.gov/pubmed/32964995) | [34014438](https://www.ncbi.nlm.nih.gov/pubmed/34014438) |
| miR-126a-3p | Malat1 | [32591985](https://www.ncbi.nlm.nih.gov/pubmed/32591985) |  |
| miR-126a-3p | Neat1 | [33677813](https://www.ncbi.nlm.nih.gov/pubmed/33677813) |  |
| miR-126a-3p | Pvt1 | [29277611](https://www.ncbi.nlm.nih.gov/pubmed/29277611) |  |
| miR-143-3p | H19 | [33758383](https://www.ncbi.nlm.nih.gov/pubmed/33758383) | [30551533](https://www.ncbi.nlm.nih.gov/pubmed/30551533) |
| miR-143-3p | Malat1 | [33564242](https://www.ncbi.nlm.nih.gov/pubmed/33564242) | [29741283](https://www.ncbi.nlm.nih.gov/pubmed/29741283) |
| miR-143-3p | Mir22hg | predicted |  |
| miR-143-3p | Pvt1 | [30825877](https://www.ncbi.nlm.nih.gov/pubmed/30825877) |  |
| miR-146a-5p | Dancr | predicted |  |
| miR-146a-5p | Neat1 | [31484042](https://www.ncbi.nlm.nih.gov/pubmed/31484042) |  |
| miR-148a-3p | H19 | [30362572](https://www.ncbi.nlm.nih.gov/pubmed/30362572) | [31934270](https://www.ncbi.nlm.nih.gov/pubmed/31934270) |
| miR-148a-3p | Meg3 | [24515776](https://www.ncbi.nlm.nih.gov/pubmed/24515776) |  |
| miR-148a-3p | Neat1 | [33550894](https://www.ncbi.nlm.nih.gov/pubmed/33550894) | [33011404](https://www.ncbi.nlm.nih.gov/pubmed/33011404) |
| miR-148a-3p | Pvt1 | [34288373](https://www.ncbi.nlm.nih.gov/pubmed/34288373) | [34859371](https://www.ncbi.nlm.nih.gov/pubmed/34859371) |
| miR-148a-3p | Snhg20 | [34836544](https://www.ncbi.nlm.nih.gov/pubmed/34836544) |  |
| miR-148a-3p | Snhg4 | [32525017](https://www.ncbi.nlm.nih.gov/pubmed/32525017) |  |
| miR-148a-3p | Tug1 | predicted |  |
| miR-200a-3p | H19 | [30240970](https://www.ncbi.nlm.nih.gov/pubmed/30240970) | [30137188](https://www.ncbi.nlm.nih.gov/pubmed/30137188) |
| miR-200a-3p | Neat1 | [34448644](https://www.ncbi.nlm.nih.gov/pubmed/34448644) |  |
| miR-200a-3p | Pvt1 | [28731781](https://www.ncbi.nlm.nih.gov/pubmed/28731781) |  |
| miR-378a-3p | Malat1 | [34674599](https://www.ncbi.nlm.nih.gov/pubmed/34674599) |  |
| miR-378a-3p | Neat1 | [31904498](https://www.ncbi.nlm.nih.gov/pubmed/31904498) |  |
| miR-378a-3p | Snhg1 | predicted |  |
| miR-378a-3p | Snhg15 | predicted |  |
| miR-378a-3p | Tug1 | predicted |  |
